# Factors Associated With Extended Resection (Cecal Resection or Right Hemicolectomy) in Patients With Acute Appendicitis at a Tertiary Private Hospital: A Retrospective Cohort Study

**DOI:** 10.7759/cureus.119274

**Published:** 2026-11-07

**Authors:** Cielo Scarlet Silva-Ramos, Jorge Luis Leal-Hidalgo, Sara Fernanda Arechavala-Lopez, Maria Fernanda Ramos-Griera, Kevin Joseph Fuentes-Calvo, Alejandro Alfaro-Goldaracena, Alejandro Díaz Girón-Gidi, Eduardo Villegas-Tovar, Eduardo Esteban Montalvo-Jave

**Affiliations:** 1 General Surgery, Facultad Mexicana de Medicina, Universidad La Salle México, Mexico City, MEX; 2 General Surgery, Hospital Médica Sur, Mexico City, MEX; 3 Medicine, Hospital Médica Sur, Mexico City, MEX; 4 Surgery, National Institute for Medical Sciences and Nutrition Salvador Zubiran, Mexico City, MEX; 5 General and Gastrointestinal Surgery, Angeles Health System, Mexico City, MEX; 6 General Surgery, Facultad de Medicina, Universidad Nacional Autónoma de México (UNAM), Mexico City, MEX

**Keywords:** acute appendicitis, complicated appendicitis, extended resection, right hemicolectomy, risk factors

## Abstract

Introduction: Acute appendicitis may require surgical procedures beyond conventional appendectomy, including cecal resection and right hemicolectomy. This study aimed to identify clinical factors associated with extended resection in adults undergoing surgery for acute appendicitis.

Methods: This retrospective, single-center cohort study included 360 adults treated surgically for acute appendicitis at a tertiary care hospital between 2018 and 2023. Extended resection was defined as cecal resection or right hemicolectomy. Associations were assessed using univariate analyses and multivariable logistic regression adjusted for age, symptom duration, previous abdominal surgery, diabetes mellitus, and hypertension. Appendicitis severity was evaluated in a secondary model using grouped phases. Missing data were not imputed. The study received ethics committee approval (2024-EXT-871).

Results: Extended resection occurred in 50 of 351 patients with available outcome data (14.2%). Longer symptom duration (adjusted odds ratio (aOR) 1.185; 95% confidence interval (CI) 1.004-1.398; p=0.045) and older age (aOR 1.022; 95% CI 1.001-1.045; p=0.045) were associated with extended resection in the primary model. In the secondary model, advanced appendicitis phases were strongly associated with extended resection (aOR 10.273; 95% CI 4.694-22.481; p<0.001).

Conclusion: Longer symptom duration was associated with extended resection, including after adjustment for grouped appendicitis severity. These findings warrant cautious interpretation given the retrospective design and limited number of events.

## Introduction

Acute appendicitis is one of the most common surgical emergencies worldwide and represents an important cause of acute abdominal pain requiring surgical evaluation [1,2]. Although its clinical presentation and progression vary among patients, accurate diagnosis and appropriate treatment remain essential for preventing disease-related complications.

Appendectomy is the standard surgical treatment for acute appendicitis. However, complicated cases may present substantial challenges because of extensive inflammation, tissue destruction, or involvement of adjacent anatomical structures. In selected patients, inflammatory cecal masses may resemble acute appendicitis and require more extensive surgical procedures, including right hemicolectomy [3]. The management of complicated appendicitis may also involve initial nonoperative treatment in appropriately selected patients, followed by interval appendectomy when indicated [4].

The definition of complicated appendicitis varies across studies and clinical classifications. It commonly encompasses perforated appendicitis, appendiceal abscess, and inflammatory phlegmon, while some definitions also include gangrenous or necrotic appendicitis. These manifestations should be distinguished from the intraoperative classification used in the present study, which describes appendicitis according to four phases of inflammatory severity. In particular, phlegmonous inflammation of the appendiceal wall does not necessarily indicate the presence of a periappendiceal inflammatory mass or abscess.

In patients with extensive inflammatory involvement of the appendiceal base or adjacent cecal tissue, conventional appendectomy may not be sufficient or technically feasible. Under these circumstances, cecal resection or right hemicolectomy may be necessary, depending on the anatomical extent of disease, tissue viability, and intraoperative findings. Previous investigations have examined preoperative factors associated with extended resection in complicated appendicitis, including symptom duration and disease-related characteristics [5]. Other surgical reports have described more extensive colonic resections in different clinical settings, emphasizing that operative indications and anatomical considerations vary substantially between patients [6].

Cecal resection and right hemicolectomy differ in anatomical extent, indications, and potential postoperative consequences. Moreover, extended right hemicolectomy has also been investigated in patients with malignant colonic disease, a clinical setting that differs fundamentally from acute appendicitis [7]. Therefore, findings from oncological resections should not be directly extrapolated to patients undergoing surgery for inflammatory appendiceal disease. In the present study, cecal resection and right hemicolectomy were grouped under the operational definition of extended resection because both represent intestinal resections beyond conventional appendectomy. This combined outcome was selected to investigate clinical factors associated with the occurrence of additional intestinal resection, rather than to establish surgical equivalence or compare postoperative outcomes between procedures.

The timing of surgical intervention is another relevant consideration in appendicitis management. Previous research has investigated the relationship between time to appendectomy and the risk of perforation [8]. However, symptom duration before presentation and the interval between hospital admission and surgery represent distinct clinical variables. Consequently, an association between prolonged symptoms and extended resection should not be interpreted as evidence that earlier surgical intervention would necessarily prevent more extensive surgery.

The objective of the present study was to evaluate clinical factors associated with extended intestinal resection, defined as cecal resection or right hemicolectomy, among adult patients undergoing surgical treatment for acute appendicitis at a tertiary-care hospital.

## Materials and methods

Study design and setting

This single-center, retrospective observational study was conducted at Hospital Médica Sur, a tertiary care private hospital in Mexico City, Mexico. The study aimed to identify clinical and perioperative factors associated with extended intestinal resection among patients undergoing surgical treatment for acute appendicitis. The study period covered 2018-2023, as established in the original research protocol.

Study population and eligibility criteria

The study population consisted of patients registered in the institutional surgical database who underwent operative management for acute appendicitis during the study period. The target population comprised adult patients aged 18 years or older with a clinical diagnosis of acute appendicitis who underwent surgical treatment. A total of 360 records were available in the analytical database. Records with missing information for individual variables were retained for descriptive analyses, and the number of observations included in each statistical analysis depended on data availability. Patients with missing primary outcome data were excluded only from analyses requiring that outcome.

Data collection and study variables

Demographic, clinical, laboratory, imaging, and perioperative information was obtained retrospectively from the available institutional database. Demographic and clinical variables included age, sex, body mass index (BMI), diabetes mellitus, hypertension, immunosuppression, history of cancer, previous abdominal surgery, symptom duration, and documented fever. Laboratory variables included leukocyte count, neutrophil count, C-reactive protein, and procalcitonin. Imaging information included appendiceal diameter measured by computed tomography (CT), when available. Operative variables included surgical approach, conversion to open surgery, cecal resection, right hemicolectomy, operative duration, intraoperative blood loss, and postoperative drain placement. Symptom duration was analyzed as a continuous variable expressed in days, according to the information recorded in the database.

Classification of acute appendicitis

Appendicitis severity was classified using the four-phase system described by Hernández-Orduña et al. [9]. Phase I corresponded to congestive or catarrhal appendicitis; phase II to suppurative or phlegmonous appendicitis; phase III to gangrenous or necrotic appendicitis; and phase IV to perforated appendicitis. The phase documented in the surgical database was used for statistical analysis. Patients without a recorded phase were classified as having missing information for this variable. Because appendicitis phase was documented in the operative setting, it was considered an intraoperative severity characteristic rather than an independently established preoperative predictor.

Definition of extended intestinal resection

The primary outcome was extended intestinal resection performed during surgical management of acute appendicitis. Extended resection was defined as cecal resection or right hemicolectomy, according to the operative procedure documented in the database. Cecal resection was considered a limited resection involving the cecum, whereas right hemicolectomy represented a more extensive resection of the right colon. The recorded operative procedure was used to determine the primary outcome. Patients with an explicitly negative extended-resection outcome were classified as not having undergone extended resection. Missing outcome values were retained as missing and were not recoded as negative.

Histopathological information and alternative diagnoses

Extended intestinal resection was defined according to the operative procedure documented in the institutional database, independently of the final histopathological diagnosis. Histopathological findings were not systematically available in the analytical dataset and therefore could not be evaluated as a separate variable. Although a history of cancer was recorded, the database did not allow differentiation between pre-existing malignancies, incidental appendiceal neoplasms, or neoplastic disease potentially contributing to the decision to perform extended resection. Consequently, the potential influence of underlying neoplastic disease on the primary outcome could not be determined.

Statistical analysis

Continuous variables were summarized as means and standard deviations (SDs) or medians and interquartile ranges (IQRs), as appropriate. Categorical variables were summarized as absolute frequencies and percentages. Percentages were calculated using the number of patients with available information for each variable. For univariate comparisons between patients with and without extended resection, Welch’s independent-samples t-test was used for age and BMI. The Mann-Whitney U test was used for symptom duration, leukocyte count, neutrophil count, C-reactive protein, procalcitonin, appendiceal diameter on CT, operative duration, and intraoperative blood loss. Categorical variables were compared using Pearson’s chi-square (χ²) test when expected cell frequencies were adequate. Fisher’s exact test was used for sparse 2 × 2 contingency tables. The association between the four appendicitis phases and extended resection was evaluated using Pearson’s χ² test. Test statistics, degrees of freedom when applicable, and two-sided p-values were reported.

A multivariable binary logistic regression model was fitted to evaluate the associations of symptom duration, age, previous abdominal surgery, diabetes mellitus, and hypertension with extended resection. These variables were selected based on clinical relevance and their consideration in the original study analyses rather than exclusively on univariate statistical significance. Adjusted odds ratios (aORs), 95% confidence intervals (CIs), and p-values were calculated. Because no extended resection events were observed among patients with phase I appendicitis, the original phase-specific logistic regression model using phase I as the reference category was not retained. An exploratory sensitivity analysis was performed by grouping appendicitis severity into phases I-II and III-IV and incorporating this dichotomous variable into the multivariable logistic regression model. This secondary analysis was intended to evaluate the robustness of the association between symptom duration and extended resection after accounting for operative severity, while avoiding the zero-event reference category observed in phase I. The sensitivity analysis was interpreted cautiously because appendicitis severity was documented in the operative setting and may be closely related to the surgical decision itself.

Missing values were not imputed. Descriptive and univariate analyses were performed using available observations for each variable. Multivariable analyses were conducted using complete cases for the variables included in each model. All statistical tests were two-sided. A p-value below 0.05 was considered statistically significant. Given the exploratory nature of the study and the multiple comparisons performed, results were interpreted cautiously.

## Results

Study population and data availability

The available database contained 360 patient records. Information on the primary outcome was available for 351 patients, of whom 50 (14.2%) underwent extended intestinal resection, and 301 (85.8%) did not. Nine records had missing information on the primary outcome. Data availability varied across study variables. Age and sex were available for all 360 records, whereas BMI and symptom duration were available for 359 patients. Appendicitis phase was documented in 314 patients. C-reactive protein and procalcitonin measurements were available for 70 and 10 patients, respectively. The demographic, clinical, laboratory, and perioperative characteristics of the study population are summarized in Table 1.

**Table 1:** Demographic, clinical, laboratory, and perioperative characteristics of the study population (n = 360). Values are presented as mean ± standard deviation (SD), median (interquartile range (IQR)), or number (percentage). Percentages were calculated using the number of patients with available data for each variable. Missing observations were not imputed. CT: computed tomography; BMI: body mass index

Variable	Result	Available (n)
Age, years, mean ± SD	41.11 ± 15.89	360
Female sex, n (%)	192 (53.3)	360
Male sex, n (%)	168 (46.7)	360
BMI, kg/m², mean ± SD	25.79 ± 4.27	359
Diabetes mellitus, n (%)	17 (4.7)	360
Hypertension, n (%)	36 (10.0)	360
Previous abdominal surgery, n (%)	115 (32.0)	359
Immunosuppression, n (%)	4 (1.1)	359
History of cancer, n (%)	9 (2.5)	359
Symptom duration, days, median (IQR)	1 (0–1)	359
Documented fever, n (%)	44 (43.6)	101
Leukocyte count, ×10³/µL, mean ± SD	12.70 ± 4.22	355
Neutrophil count, ×10³/µL, mean ± SD	10.42 ± 4.19	341
C-reactive protein, median (IQR)	25.38 (5.58–124.90)	70
Procalcitonin, median (IQR)	0.155 (0.044–1.813)	10
Appendiceal diameter on CT, cm, median (IQR)	1.2 (1.0–1.5)	270
Operative duration, min, median (IQR)	60 (45–70)	332
Intraoperative blood loss, mL, median (IQR)	15 (10–20)	318
Postoperative drain placement, n (%)	72 (20.5)	351
Appendicitis phase I, n (%)	46 (14.6)	314
Appendicitis phase II, n (%)	166 (52.9)	314
Appendicitis phase III, n (%)	49 (15.6)	314
Appendicitis phase IV, n (%)	53 (16.9)	314
Extended resection, n (%)	50 (14.2)	351

Demographic and clinical characteristics

The mean age of the study population was 41.11 ± 15.89 years, and 192 patients (53.3%) were female. The mean BMI was 25.79 ± 4.27 kg/m². Diabetes mellitus was documented in 17 patients (4.7%), hypertension in 36 (10.0%), previous abdominal surgery in 115 (32.0% of patients with available information), and a history of cancer in nine (2.5% of patients with available information). The median recorded symptom duration was one day (IQR, 0-1). Appendicitis phase was available for 314 patients. Of these, 46 (14.6%) were classified as phase I, 166 (52.9%) as phase II, 49 (15.6%) as phase III, and 53 (16.9%) as phase IV.

Univariate analysis

Patients undergoing extended resection were older than those without extended resection (45.70 ± 16.29 versus 40.15 ± 15.65 years; Welch’s t-test, t = −2.244, p = 0.028). Symptom duration differed significantly between groups. Although the median duration was one day in both groups, the IQR was 1-1.75 days in the extended resection group and 0-1 day in the non-extended resection group (Mann-Whitney U = 5,981.0; p = 0.012). No statistically significant differences were identified for sex (p = 0.444), BMI (p = 0.679), diabetes mellitus (p = 0.245), hypertension (p = 0.797), previous abdominal surgery (p = 0.395), immunosuppression (p = 1.000), or history of cancer (p = 0.123). Among laboratory and imaging variables, leukocyte count (p = 0.081), neutrophil count (p = 0.098), C-reactive protein (p = 0.097), and appendiceal diameter on CT (p = 0.222) did not differ significantly between groups. Procalcitonin was available for only nine patients with a documented primary outcome. Although the comparison was not statistically significant (p = 1.000), the small number of observations limited its interpretation. Operative duration was longer among patients undergoing extended resection, with a median of 60 minutes (IQR, 55-80), compared with 55 minutes (IQR, 45-70) among those without extended resection (Mann-Whitney U = 4,907.0; p = 0.003). Intraoperative blood loss was also greater in the extended resection group, with a median of 20 mL (IQR, 13.75-50), compared with 10 mL (IQR, 10-20) in the non-extended resection group (Mann-Whitney U = 4,151.5; p < 0.001). Postoperative drain placement was more frequent among patients undergoing extended resection (29/50, 58.0%) than among those without extended resection (43/300, 14.3%; Pearson’s χ² = 50.013; p < 0.001). These operative characteristics were considered descriptive findings associated with the surgical procedure rather than preoperative predictors of extended resection (Table 2).

**Table 2:** Univariate comparison of patients with and without extended intestinal resection. Values are presented as mean ± SD, median (IQR), or n/N (%). Welch’s independent-samples t-test was used for age and BMI. The Mann–Whitney U test was used for symptom duration, laboratory measurements, appendiceal diameter, operative duration, and blood loss. Pearson’s chi-square (χ²) test was used for categorical comparisons with adequate expected cell frequencies, and Fisher’s exact test was used for sparse 2 × 2 comparisons. All tests were two-sided. For Mann–Whitney U tests, the non-extended resection group was designated as the first comparison group. Denominators reflect available observations for each variable.

Variable	No extended resection	Extended resection	Statistical test	p-value
Age, years, mean ± SD	40.15 ± 15.65 (n=301)	45.70 ± 16.29 (n=50)	Welch’s t-test t(64.93) = −2.244	0.028
Female sex, n/N (%)	157/301 (52.2)	29/50 (58.0)	Pearson χ² χ²(1) = 0.587	0.444
BMI, kg/m², mean ± SD	25.86 ± 4.28 (n=300)	25.59 ± 4.23 (n=50)	Welch’s t-test t(66.82) = 0.416	0.679
Diabetes mellitus, n/N (%)	11/301 (3.7)	4/50 (8.0)	Fisher’s exact	0.245
Hypertension, n/N (%)	28/301 (9.3)	5/50 (10.0)	Fisher’s exact	0.797
Previous abdominal surgery, n/N (%)	90/300 (30.0)	18/50 (36.0)	Pearson χ² χ²(1) = 0.723	0.395
Immunosuppression, n/N (%)	3/300 (1.0)	0/50 (0.0)	Fisher’s exact	1
History of cancer, n/N (%)	6/300 (2.0)	3/50 (6.0)	Fisher’s exact	0.123
Symptom duration, days, median (IQR)	1 (0–1), n=300	1 (1–1.75), n=50	Mann–Whitney U U = 5981.0	0.012
Documented fever, n/N (%)	34/81 (42.0)	5/13 (38.5)	Fisher’s exact	1
Leukocyte count, ×10³/µL, median (IQR)	12.3 (9.6–14.88), n=298	13.3 (10.0–17.0), n=49	Mann–Whitney U U = 6164.5	0.081
Neutrophil count, ×10³/µL, median (IQR)	10.3 (7.4–12.8), n=285	11.15 (8.08–13.95), n=48	Mann–Whitney U U = 5818.0	0.098
C-reactive protein, median (IQR)	24.52 (3.49–77.76), n=51	116.14 (19.26–177.75), n=16	Mann–Whitney U U = 294.5	0.097
Procalcitonin, median (IQR)	0.22 (0.04–25.72), n=7	0.14 (0.10–0.19), n=2	Mann–Whitney U U = 7.0	1
Appendiceal diameter on CT, cm, median (IQR)	1.2 (1.0–1.5), n=231	1.3 (1.0–1.6), n=36	Mann–Whitney U U = 3634.5	0.222
Operative duration, min, median (IQR)	55 (45–70), n=284	60 (55–80), n=47	Mann–Whitney U U = 4907.0	0.003
Intraoperative blood loss, mL, median (IQR)	10 (10–20), n=269	20 (13.75–50), n=48	Mann–Whitney U U = 4151.5	<0.001
Postoperative drain placement, n/N (%)	43/300 (14.3)	29/50 (58.0)	Pearson χ² χ²(1) = 50.013	<0.001
Appendicitis phase	No extended resection (n=267)	Extended resection (n=45)		
Phase I	46 (17.2%)	0 (0.0%)		
Phase II	156 (58.4%)	10 (22.2%)		
Phase III	37 (13.9%)	11 (24.4%)		
Phase IV	28 (10.5%)	24 (53.3%)		
Overall comparison	Pearson χ²(3) = 62.464	p < 0.001		

Appendicitis severity

Among the 312 patients with both appendicitis phase and primary outcome documented, extended resection occurred in 0 of 46 patients with phase I appendicitis, 10 of 166 with phase II, 11 of 48 with phase III, and 24 of 52 with phase IV. The distribution of appendicitis phases differed significantly between patients with and without extended resection (Pearson’s χ² = 62.464; degrees of freedom = 3; p < 0.001).

Multivariable analysis

The primary multivariable logistic regression model included 349 patients with complete data for the primary outcome and the selected covariates. Fifty patients in this model underwent extended resection. Longer symptom duration was associated with greater odds of extended resection (aOR, 1.185 per additional day; 95% CI, 1.004-1.398; p = 0.045). Older age was also associated with greater odds of extended resection (aOR, 1.022 per additional year; 95% CI, 1.001-1.045; p = 0.045). Previous abdominal surgery (aOR, 0.998; 95% CI, 0.501-1.990; p = 0.996), diabetes mellitus (aOR, 2.454; 95% CI, 0.685-8.788; p = 0.168), and hypertension (aOR, 0.447; 95% CI, 0.134-1.495; p = 0.191) were not significantly associated with extended resection (Table 3).

**Table 3:** Multivariable logistic regression analysis of factors associated with extended intestinal resection (n = 349; extended resection events = 50) Multivariable binary logistic regression was performed using complete cases. The model included symptom duration, age, previous abdominal surgery, diabetes mellitus, and hypertension. Adjusted odds ratios (aORs), 95% confidence intervals (CIs), and two-sided p-values are reported. The analysis included 349 patients and 50 extended resection events.

Variable	Adjusted OR	95% CI	p-value
Symptom duration, per additional day	1.185	1.004–1.398	0.045
Age, per additional year	1.022	1.001–1.045	0.045
Previous abdominal surgery	0.998	0.501–1.990	0.996
Diabetes mellitus	2.454	0.685–8.788	0.168
Hypertension	0.447	0.134–1.495	0.191

Exploratory sensitivity analysis

An additional multivariable logistic regression model incorporated appendicitis severity as a dichotomous variable comparing phases III-IV with phases I-II. This analysis included 311 patients with complete data, of whom 45 underwent extended resection. Advanced appendicitis phase was associated with greater odds of extended resection (aOR, 10.273; 95% CI, 4.694-22.481; p < 0.001). Symptom duration remained associated with the outcome after adjustment for appendicitis severity and the other covariates (aOR, 1.204 per additional day; 95% CI, 1.003-1.447; p = 0.047). Age (p = 0.561), previous abdominal surgery (p = 0.762), diabetes mellitus (p = 0.713), and hypertension (p = 0.491) were not statistically significant in this secondary model (Table 4). Because appendicitis severity was documented in the operative setting, its association with extended resection should not be interpreted as evidence of independent preoperative predictive ability.

**Table 4:** Exploratory multivariable logistic regression including grouped appendicitis severity ( n = 311; extended resection events = 45) Exploratory multivariable logistic regression using complete cases. Appendicitis severity was grouped as phases I–II versus phases III–IV to avoid the zero-event reference category present in the original phase-specific model. Because severity was documented in the operative setting, the association should not be interpreted as independent preoperative predictive performance. OR: odds ratio; CI: confidence interval

Variable	Adjusted OR	95% CI	p-value
Symptom duration, per additional day	1.204	1.003–1.447	0.047
Age, per additional year	1.008	0.982–1.034	0.561
Previous abdominal surgery	0.884	0.399–1.958	0.762
Diabetes mellitus	1.295	0.326–5.141	0.713
Hypertension	0.633	0.172–2.329	0.491
Appendicitis phases III–IV versus I–II	10.273	4.694–22.481	<0.001

## Discussion

In this retrospective cohort of patients undergoing surgical treatment for acute appendicitis, longer symptom duration was associated with increased odds of extended intestinal resection. Among the 351 patients with available outcome data, 50 (14.2%) underwent extended resection. In the primary multivariable analysis, each additional day of symptoms was associated with an 18.5% increase in the odds of extended resection (aOR 1.185, 95% CI 1.004-1.398; p = 0.045). Age was also associated with the outcome (aOR 1.022 per year, 95% CI 1.001-1.045; p = 0.045). However, the CIs for both associations approached the null value, indicating that these findings should be interpreted cautiously. Previous studies have similarly identified prolonged symptom duration as a factor associated with the need for more extensive intestinal resection in complicated appendicitis [5].

The frequency of extended resection in our cohort (14.2%) was higher than the 6.7% reported by Kobayashi et al. [10] and the 11.5% reported by Waki et al. [5]. These differences may reflect variations in patient selection, disease severity, institutional referral patterns, and operative indications. Kobayashi et al. identified prolonged symptom duration, retrocecal or retrocolic appendix location, and abscess formation as factors associated with extended resection [10]. Similarly, Waki et al. reported an association between prolonged symptom duration and the need for extended resection in complicated appendicitis [5]. Although these findings are consistent with the direction of the association observed in our study, differences in study populations and outcome definitions limit direct comparisons. Moreover, the observed association between symptom duration and extended resection should not be interpreted as evidence that a specific duration threshold determines the need for more extensive surgery.

Appendicitis severity was strongly associated with extended resection. No extended resections occurred among patients classified as phase I, whereas the frequency increased in more advanced phases. In the exploratory sensitivity analysis, grouping phases III-IV and comparing them with phases I-II demonstrated a substantial association between advanced appendicitis severity and extended resection (adjusted OR 10.273, 95% CI 4.694-22.481; p < 0.001). Symptom duration remained associated with the outcome after adjustment for grouped severity (adjusted OR 1.204 per additional day, 95% CI 1.003-1.447; p = 0.047). However, appendicitis phase was documented intraoperatively and reflects anatomical findings that may also influence the decision to perform extended resection. Therefore, these findings should not be interpreted as establishing the independent preoperative predictive value of appendicitis severity.

The relationship between symptom duration and extended resection must be distinguished from the timing of surgical intervention after hospital admission. Our study evaluated symptom duration but did not specifically assess in-hospital waiting time or compare immediate surgery with delayed operative management. Previous studies have examined the relationship between operative timing and outcomes in complicated appendicitis, including the potential role of delayed surgery in selected patients [8,11]. In addition, a systematic review and meta-analysis evaluated early versus delayed appendectomy in patients with appendiceal abscess or phlegmon [12]. These findings highlight the importance of considering disease presentation and treatment selection when interpreting the association between symptom duration and extended resection. However, our results cannot establish whether earlier surgical intervention would have prevented the need for extended resection.

The decision to perform extended intestinal resection is influenced by the extent of local inflammation, tissue viability, involvement of the appendiceal base or cecum, and the feasibility of safely completing a conventional appendectomy. In selected patients with localized complicated appendicitis, particularly those presenting with an appendiceal abscess or phlegmon, initial nonoperative management may be considered according to clinical circumstances [4,12]. Nevertheless, our study did not evaluate the comparative effectiveness of operative and nonoperative strategies, and symptom duration alone should not be interpreted as an indication for extended resection.

The classification of appendicitis severity used in our study provided a standardized description of intraoperative disease progression. However, different classification systems capture distinct anatomical and clinical characteristics. Previous studies have evaluated the association between anatomical severity and postoperative outcomes using the American Association for the Surgery of Trauma grading system [13]. Similarly, Gomes et al. proposed a comprehensive grading system incorporating clinical, imaging, and laparoscopic findings, including specific consideration of appendiceal-base necrosis [14]. Although these classifications provide relevant information regarding appendicitis severity, our study did not directly compare their predictive performance or evaluate whether individual anatomical characteristics independently predicted extended resection. Consequently, no conclusions regarding the superiority of one classification system over another can be drawn from our findings.

Diabetes mellitus, hypertension, and previous abdominal surgery were not statistically significantly associated with extended resection in the primary multivariable analysis. However, the confidence intervals were wide and included potentially clinically relevant effects. Therefore, the absence of statistical significance should not be interpreted as evidence that these conditions have no association with the need for extended resection. Previous studies have examined clinical factors associated with complications and treatment outcomes in adult appendicitis [15]. Nevertheless, differences in study populations, outcomes, and analytical approaches limit direct comparisons with our findings. Furthermore, although age was statistically significant in the primary multivariable model, this association was not statistically significant in the exploratory model incorporating grouped appendicitis severity. These findings underscore the uncertainty surrounding the independent contribution of individual clinical characteristics.

Extended intestinal resection encompasses procedures that differ in anatomical extent and potential postoperative implications. In our cohort, 45 patients underwent cecal resection and five underwent right hemicolectomy. Previous research has compared ileocecal resection with right hemicolectomy in patients requiring extended surgery for complicated appendicitis [16]. However, our study was not designed to compare these procedures, and the small number of right hemicolectomies precluded a meaningful procedure-specific analysis. In addition, postoperative complications, length of hospital stay, and long-term outcomes were not systematically available. Therefore, our findings cannot establish whether one surgical approach was associated with more favorable postoperative outcomes.

This study has several limitations. First, its retrospective, single-center design introduces the possibility of selection bias and limits the generalizability of the findings. Second, the relatively small number of extended resection events (n = 50) restricted the precision of multivariable estimates, particularly for less prevalent clinical characteristics. Third, multiple univariate comparisons were performed without adjustment for multiplicity, and these findings should be interpreted as exploratory. Fourth, missing information affected several variables, including appendicitis phase, potentially introducing bias through complete-case analysis.

Additional limitations concern the available clinical and surgical information. The study period encompassed the COVID-19 pandemic, which may have influenced healthcare access and patterns of presentation, although these effects were not directly evaluated. Surgical approach, surgeon experience, and specific intraoperative indications for extended resection were not systematically analyzed. Histopathological findings were not consistently available, preventing assessment of the potential contribution of incidental neoplasia to surgical decision-making. Furthermore, the absence of systematic postoperative follow-up prevented evaluation of complications and long-term outcomes. Finally, the absence of extended resection events among patients with phase I appendicitis limited conventional phase-specific logistic regression. Although the exploratory sensitivity analysis using grouped severity addressed the zero-event reference category, it did not eliminate the limitations associated with sparse data and model specification.

Despite these limitations, our findings suggest that symptom duration may provide clinically accessible information associated with the likelihood of extended intestinal resection in patients undergoing surgery for acute appendicitis. However, its interpretation should consider the uncertainty of the estimated associations, the severity of appendicitis, and the intraoperative anatomical findings that ultimately determine the extent of surgery.

## Conclusions

In this retrospective analysis of patients undergoing surgery for acute appendicitis, longer symptom duration was associated with increased odds of extended resection. This association was also observed in an exploratory analysis incorporating appendicitis severity. However, given the retrospective design and the uncertainty surrounding the estimates, these findings should be interpreted cautiously. Age also showed a statistically significant association in the primary model; however, this finding was not maintained in the exploratory analysis incorporating grouped appendicitis severity. The CIs for symptom duration approached the null value, warranting cautious interpretation.

Advanced appendicitis severity was strongly associated with extended resection in the exploratory analysis, although its intraoperative assessment limits its applicability for preoperative prediction. Cecal resection and right hemicolectomy were evaluated as a combined outcome rather than compared as separate surgical procedures. Consequently, this study cannot establish differences in their indications or postoperative outcomes. Future prospective studies should evaluate these procedures separately, incorporating operative indications, postoperative complications, and long-term outcomes. Overall, symptom duration may provide useful clinical information when anticipating the possibility of extended resection, but the definitive surgical approach must be guided by individual patient characteristics and intraoperative anatomical findings.
